# Supplementary material for: PLEKHM2 Loss of Function Impairs the Activity of iPSC-Derived Neurons via Regulation of Autophagic Flux
Source: Int J Mol Sci. 2022 Dec 17;23(24):16092. doi: 10.3390/ijms232416092 (PMC9782635; doi:10.3390/ijms232416092)
Supplement: Supplementary file 1 [file ijms-23-16092-s001.zip › ijms-1997908-supplementary.pdf]

International Journal of
*Molecular Sciences*

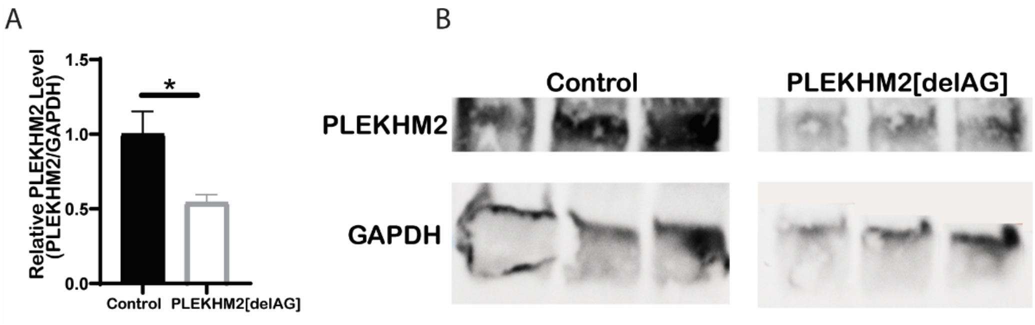


**Supplementary Figure S1.** PLEKHM2 expression in neuroepithelial (NEP) cultures. **A,** Western blot analysis. **B,** Representative western blot membranes of control and PLEKHM2[delAG]. Two tailed student's T-test was used * $p < 0.05$, presenting mean ± SEM, control n=5, PLEKHM2[delAG] n=4.

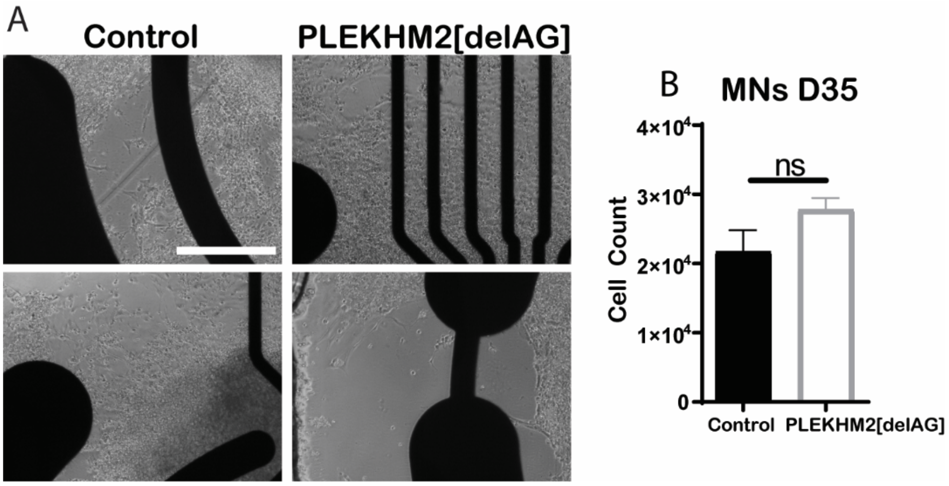


**Supplementary Figure S2.** **E**stimation of cell number at the end point of the experiment, observing MEA cultured cell populations **A** Representative images of MEA cultures at the end point, D35 before performing a flow cytometry assay. Cell density did not significantly vary between cultures. Scale bar - 400μm **B** Cell numbers within the gate in flow cytometry analysis. Two-tailed student's t-test was employed $p = 0.1139$, presenting mean ± SEM n=6.
